# Quality of life in Brazilian elderly: an analysis of healthy aging from the perspective of Potter's global bioethics

**DOI:** 10.1080/11287462.2021.1966975

**Published:** 2021-08-19

**Authors:** Olivia Figueira, Helena Figueira, Renato Soleiman Franco, Paulo Sergio Marcellini, Anor Sganzerla, Carla Corradi Perini

**Affiliations:** a Graduate Program in Bioethics at the Pontifical Catholic University of Paraná (PUCPR), Parana, Brazil; b UNIRIO-RJ, Rio de Janeiro, Brazil

**Keywords:** Quality of life, bioethics, elderly, healthy aging, global bioethics‌

## Abstract

**Introduction:**

Quality of Life (QOL) is essential for healthy aging and through the WHOQOL-Old, it is possible to analyze factors that increase vulnerability and reduce QOL. Aligned with healthy aging is Potter's global bioethics proposing expanded ethics and social justice.

**Objective:**

To analyze the QOL of Brazilian elderly from the perspective of Potteŕs global bioethics.

**Method:**

Analytical observational research with a quantitative approach composed of 280 Brazilian, aged 60 or over, of both gender, volunteers, who answered the WHOQOL-Old online.

**Result:**

Global score of 77.9%, with the mean ± standard deviation: Functioning of the senses 86% (17.22 ± 2.80); Autonomy 78.5% (15.7 ± 2.60); Past, present, and future activities 77.3% (15.46 ± 2.34); Social participation 74.9% (14.99 ± 2.62); Death and dying 71.6% (14.33 ± 3.88) and Intimacy 79.1% (15.82 ± 2.82).

**Conclusion:**

Elderly perceived their QOL positively. In the quest to promote healthy aging, it is necessary to broaden the vision for social justice proposed by Potteŕs global bioethics.

## Introduction

According to the United Nations (UN), by 2050 the world's elderly population will be 2 billion people, considering elderly anyone over 60 years old. In Brazil, the elderly population once was 1 in 10 inhabitants, but this is estimated to change drastically, and in 2060 it is expected that 1 in 3 inhabitants are over 60 years old (V. H. S. Ferreira et al., 2020). Brazil is the fifth most populous country in the world and has experienced one of the fastest aging processes, a trend that tends to accelerate in this century. In Brazil, we also find one of the largest socioeconomic discrepancies between classes, and therefore, populational aging has occurred within this context of extreme inequality (Lima-Costa et al., 2018).

With populational aging increasing exponentially worldwide, it is necessary to know the various factors that can influence the Quality of Life (QOL) of the elderly, the same being essential for healthy aging. To measure this QOL, the WHOQOL-Old questionnaire was created and validated internationally, consisting of 24 items attributed to six facets: sensory functioning; autonomy; past, present, and future activities; social participation; death and dying; and intimacy(OMS, 2005). High QOL is associated with healthy aging, and low QOL with increased vulnerability to which elderly feel exposed, thus, QOL's self-analysis could serve as a basis for public policies, programs and actions that aim to promote healthy aging.

Vulnerability is understood as a substantive principle common to every human being, based on its intrinsic fragility. In the context of bioethical reflection, the concept of vulnerability is considered from three perspectives: vulnerability as a universal human condition, which considers that the human being is vulnerable, like every living being; vulnerability as a particular characteristic of people and groups; and vulnerability as an international bioethical principle, as proposed in the Universal Declaration on Bioethics and Human Rights (DUBDH) (Pessini, 2017). The vulnerability of the elderly is also related to biological and social factors of aging, which is often linked to the development of chronic diseases and sensory impairments, with loss of autonomy, absence of life goals and feeling of not belonging. Such relationships demonstrate the complexity and multidimensionality of vulnerability that is associated with individual and collective situations and contexts (Barbosa et al., 2019). A dynamic concept of vulnerability considers it as something multiple, and different, that can be removed one by one, as a layer. The metaphor of vulnerability layers considers that there is not only one vulnerability, as a category, but different vulnerabilities in different layers that are present simultaneously (Luna, 2004). Reducing the vulnerabilities of the elderly throughout the aging process is essential for achieving high levels of QOL.

The analysis of the QOL is considered subjective and multidimensional, being defined by the World Health Organization (WHO) as the individual's perception of his position in life, in the context of the culture and value system in which he lives, and in relation to his objectives, expectations, standards and concerns (H. A. Figueira et al., 2009). The WHO defines healthy aging as a continuous process of optimizing functionality and opportunities to maintain and improve physical and mental health, aiming to promote independence and QOL during aging (Scoralick-Lempke et al., 2018). To foster healthy aging in the long run, there is a need for structured planning, and based on this, the plan for the Decade of Healthy Aging 2021–2030 was created by the WHO Global Strategy on aging and health (OMS, 2020). The Decade of Healthy Aging established priorities that provide concrete actions based on the Madrid International Plan of Action on Aging (MIPAA) of 2002, and the WHO Global Strategy on Aging and Health, established in 2016 (Rudnicka et al., 2020).

It is important to understand the complexity of aging through theoretical frameworks, such as Van Rensselaer Potter's global bioethics, in line with scientific studies. Potter points out that the course of the planet's cultural evolution must be radically modified, and that global bioethics and world morality must be developed in the first decades of the twenty-first century (Potter & Whitehouse, 1998).

Potter proposed new ethics, combining responsibility, interdisciplinarity and interculturality, to enhance the sense of humanity (Zanella, 2018). When he launched his book Global Bioethics in 1988, he introduced a new concept, of global bioethics, with two meanings: planetary and more inclusive and comprehensive, with “macro” issues of our society and culture (Zanella et al., 2019). Potter defines a sustainable society as economically viable, socially just, culturally accepted and environmentally friendly. Thus, Potter's proposal for expanded bioethics, with a look at politics and society and aiming at sustainability, is in line with the WHO proposal for healthy aging. In this sense, this study aims to analyze the QOL and the vulnerability factors of the elderly from the perspective of Potteŕs global bioethics.

## Method

This is an observational and analytical research with a quantitative approach that took place in Brazil. The sample was characterized as finite, non-probabilistic, composed of 280 Brazilian volunteers, elderly (60 years old or more), of both gender, who agreed to participate in the research through online acceptance of the Free and Informed Consent Form in Google Forms. Data collection started on 09/22/2020 after approval by the Ethics Committee of PUCPR (CAAE: 33294420.2.0000.0020), with online sharing of the access link to the WHOQOL-Old questionnaire plus 9 sociodemographic questions in the media social groups of the researchers. Respondents invited new participants from their network of friends and acquaintances by sharing the survey link (snowball recruitment method). The collection ended on 10/06/2020 with 290 participants and 280 questionnaires fully answered.

The WHOQOL-Old Inventory consists of 24 questions divided into six facets: “Functioning of the Sensory” (FS), “Autonomy” (AUT), “Past, Present and Future Activities” (PPF), “Social Participation” (PSO), “Death and Dying” (MEM) and “Intimacy” (INT). Each facet has 4 questions, the answers can score from 4 to 20, and the higher the score, the higher the QOL (OMS, 2005). In this study, scores above 17/20 (85%) were considered high, from 15–17/20 (75–84%) were good QOL levels, and below 14/20 (74%), poor or fair. For the total, the score considered high was above 85%, good between 75–84%, fair between 50% and 74%, and poor below 49%. For the Brazilian population, the WHOQOL-Old validation study showed satisfactory characteristics of internal consistency (Cronbach coefficients from 0.71 to 0.88), discriminant validity (*p* < 0.01), concurrent validity (correlation coefficients between −0,61 and −0.50) and test-retest reliability (correlation coefficients between 0.58 and 0.82) with good psychometric performance in the investigation of QOL in the elderly (Fleck et al., 1999).

### Statistical analysis

Data analysis was performed using descriptive statistics using frequency, average, standard deviation, minimum and maximum values. From Levene's variance tests, heterogeneity was observed in some facets, opting for the Spearman correlation test. Spearman's correlation coefficient was calculated to identify the interrelation of the total QOL with the WHOQOL-Old domains (Sensory functioning, Autonomy, Past, Present, and future activities, Social participation, Death and dying, Intimacy) and total score. For comparisons, inferential statistics were used, Anova, Bonferroni and Mann–Whitney tests. All results were analyzed by Excel and SPSS 15.0, with *p* <0.05 being adopted as a statistically significant value.

## Results

The sociodemographic profile of the 280 elderly who participated in this study is shown in Table 1.

**Table 1.** Sociodemographic profile of the sample.

Questions	Answers	Absolute *n*	Percentage *n*
Gender	Female Male	210 70	75 25
Age range	60–64 65–69 70–74 75–79 80–89 >90	105 79 52 29 13 2	37.5 28.2 18.6 10.4 4.6 0.7
Who you live with	Spouse Family Alone Other	149 68 55 8	53.2 24.3 19.6 2.9
Marital status	Single Married, stable union Separated; Divorced Widower	23 172 54 31	8.2 61.4 19.3 11.1
Schooling	Illiterate E.F.I. E.F.C E.M.I. E.M.C. E.S.I. E.S.C. Graduate	0 3 6 8 33 22 84 124	0 1.1 2.1 2.9 11.8 7.9 30 44.3
Paid work	Yes No	118 162	42.1 57.9
Volunteer work	Yes No	113 150	43 57
Monthly income	1–2 S.M. 3–4 S.M. 5–6 S.M. 7 or S.M. I don't want to inform	33 46 53 104 44	11.8 16.4 18.9 37.1 15.7
Region in which you live	South Southeast Central-West Northeast North	128 95 11 28 8	47.4 35.2 4.1 10.4 3

E.F.I., Incomplete Elementary School; E.F.C., Complete Elementary School; E.M.I., Incomplete High School; E.M.C., Complete High School; E.S.I., Incomplete Higher Education; E.S.C., Complete E.S. S.M., Minimum Wage.

The Spearman Correlation, calculated to identify the interrelation of the total QOL with the facets of the WHOQOL-Old, is shown in Table 2. In the analysis performed, all facets of the WHOQOL-Old showed significant correlation, except in the autonomy and death and dying, and autonomy with intimacy.

**Table 2.** Spearman correlation of total QOL interrelation and facets.

WHOQOL-Old	TOTAL	FS	AUT	PPF	PSO	MEM	INT
TOTAL							
FS	0.606**						
AUT	0.627**	0.360**					
PPF	0.752**	0.311**	0.484**				
PSO	0.772**	0.352**	0.466**	0.728**			
MEM	0.530**	0.222**	0.075	0.192**	0.203**		
INT	0.672**	0.363**	0.389**	0.542**	0.532**	0.108	

FS, Sense functioning; AUT, autonomy; PPF, Past, Present and future activity; PSO, social participation; MEM, death and dying; INT, intimacy.

**Significant correlation at 0.01.

The elderly perceived their QOL positively, as scores above 17 (85%) were considered high, and above 15 (75%) good, and the QOL levels presented by the elderly in the sample ranged from 71.6% to 86%, as shown in Table 3.

**Table 3.** WHOQOL-Old result by facet and total score, with mean and standard deviation.

WHOQOL-Old	Average ± Standard Deviation	Average Percentage
FS	17.22 ± 2.80	86%
AUT	15.7 ± 2.60	78.5%
PPF	15.46 ± 2.34	77.3%
PSO	14.99 ± 2.62	74.9%
MEM	14.33 ± 3.88	71.6%
INT	15.82 ± 2.82	79.1%
Total	15.59 ± 1.86	77.9%

FS, Sense functioning; AUT, autonomy; PPF, Past present and future activity; PSO, social participation; MEM, death and dying; INT, intimacy.

The analysis by gender of the WHOQOL-Old results according to facet and total score are shown in Table 4, and by age group in Table 5. A difference was found between gender for death and dying (*p* = 0.001), however, there was no significant difference in the total QOL.

**Table 4.** WHOQOL-Old result by gender according to facet and total score, with mean and standard deviation.

WHOQOL-Old	Gender	Average ± Standard Deviation	Average Percentage
FS	Female Male	17.39 ± 2.66 16.74 ± 3.16	86.9% 83.7%
AUT	Female Male	15.85 ± 2.51 15.24 ± 2.82	79.2% 76.2%
PPF	Female Male	15.58 ± 2.28 15.11 ± 2.49	77.9% 75.5%
PSO	Female Male	15.12 ± 2.48 14.59 ± 2.96	75.6% 72.9%
MEM	Female Male	13.90* ± 4.00 15.64* ±3.17	69.5% 78.2%
INT	Female Male	16.00 ± 2.75 15.31 ± 2.99	80% 76.5%
TOTAL	Female Male	93.84 ± 10.35 92.64 ± 13.40	78.2% 77.2%

FS, Sense functioning; AUT, autonomy; PPF, Past present and future activity; PSO, social participation; MEM, death and dying; INT, intimacy.

**Table 5.** WHOQOL-Old result by age group according to analyzed facet and total score, mean and standard deviation.

Age group	FS	AUT	PPF	PSO	MEM	INT	TOTAL
60–64	17.23 ± 2.79	15.63 ± 2.95	15.13 ± 2.49	14.51 ± 2.62	13.86 ± 3.81	15.70 ± 3.09	92.06 ± 11.73
65–69	17.40 ± 2.57	15.74 ± 2.21	15.72 ± 1.94	15.15 ± 2.25	14.18 ± 3.74	15.81 ± 2.50	94.02 ± 9.81
70–74	17.55 ± 2.32	15.98 ± 2.07	15.63 ± 2.48	15.62 ± 2.86	14.82 ± 4.17	16.26 ± 2.25	95.88 ± 10.53
75–79	17.86 ± 2.40	16.00 ± 1.94	15.79 ± 2.21	15.59 ± 2.75	15.06 ± 4.07	16.24 ± 3.19	96.55 ± 9.35
80–89	13.69*±4.46	14.53 ± 4.29	15.38 ± 3.06	14.00 ± 2.94	15.30 ± 3.70	14.38 ± 3.59	87.30 ± 17.28
>90	15.0 ± 1.41	13.00 ± 4.24	14.50 ± 0.70	15.00 ± 1.41	15.50 ± 3.53	15.00 ± 1.41	88.00 ± 2.82

FS, Sense functioning; AUT, autonomy; PPF, Past present and future activity; PSO, social participation; MEM, death and dying; INT, intimacy.

*Significant difference *p* < 0.05, ±standard deviation.

No difference was found in the total QOL score between the different levels of education. However, a higher total QOL score and in some facets was observed in those who are married when compared to the other groups (*p* < 0.05) (Table 6).

**Table 6.** Mean comparisons test (ANOVA with Bonferroni correction) of WHOQOL-Old by marital status.

Marital status	FS	AUT	PPF	PSO	MEM	INT	TOTAL
Married; stable union	17.63 ± 2.40	15.79 ± 2.33	15.68 ± 1.97	15.28 ± 2.27	14.45 ± 3.65	16.42 ± 2.50	95.24 ± 9.75
Separated; divorced	16.34* ±3.14	15.24 ± 3.18	14.50* ±3.01	14.20* ±3.23	14.61 ± 4.18	14.53* ±2.71	89.51* ±14.26
Single	16.34 ± 2.96	15.26 ± 2.68	15.30 ± 2.16	14.17 ± 2.38	13.08 ± 4.06	14.47* ±3.04	88.65* ±10.30
Widower	17.03 ± 3.71	16.32 ± 2.82	16.09 ± 2.62	15.35 ± 3.06	14.16 ± 4.40	15.77 ± 3.04	94.74 ± 10.84

FS, Sense functioning; AUT, autonomy; PPF, Past present and future activity; PSO, social participation; MEM, death and dying; INT, intimacy.

*Significant difference *p* < 0.05 compared to married, ±standard deviation.

A higher score was found among those who live with a spouse in relation to those who live with a family (*p* < 0.05) in the total score and in most facets analyzed, except past present and future activity, and death and dying according to Table 7.

**Table 7.** Mean comparisons test (ANOVA with Bonferroni correction) of WHOQOL-Old accordind to those who live with.

Who you live with	FS	AUT	PPF	PSO	MEM	INT	TOTAL
Spouse	17.63* ±2.43	15.95* ±2.28	15.73 ± 1.99	15.45* ±2.20	14.44 ± 3.72	16.59* ±2.53	95.79* ± 9.82
Family	16.41* ±3.43	14.91* ±3.07	14.95 ± 2.49	14.09* ±2.81	14.44 ± 3.83	14.91* ±3.09	89.72* ±12.31
Other	16.12 ± 3.90	15.87 ± 3.27	13.62 ± 2.26	13.38 ± 3.46	15.25 ± 4.39	15.87 ± 1.45	90.12 ± 12.02
Alone	17.30 ± 2.52	15.94 ± 2.57	15.63 ± 2.83	15.09 ± 2.96	13.78 ± 4.31	14.89 ± 3.06	92.65 ± 11.82

FS, Sense functioning; AUT, autonomy; PPF, Past present and future activity; PSO, social participation; MEM, death and dying; INT, intimacy.

*Significant difference *p* < 0.05, ±standard deviation.

From the data and statistical analyzes demonstrated, we found that the self-analysis of the QOL of this elderly population ranges between good and high, in all facets and in the total score (from 72% to 86%) and it was found that death and dying and social participation were the facets that reduced their QOL. The elderly in the sample shows a significant correlation between the total score and almost all facets in the Spearman correlation (except autonomy & death and dying and autonomy & intimacy). Regarding the marital situation, a higher total QOL score was found in those who are married when compared to those separated / divorced (*p* < 0.05), as well as in all aspects analyzed, except death and dying. Regarding income, those who reported receiving 1–2 minimum wages have a lower score in the sensor functioning when compared to those with higher income, despite not having significantly affected the total QOL.

## Discussion

The elderly cannot be considered a homogeneous group or a homogeneous population, due to a variety of factors that influence how people age, like gender, social, economic, and cultural factors. All these factors can have a great influence in QOL and can have a narrow association with healthy aging. In Brazil, developing country, with a very unequal distribution of wealth, we can observe very significant economic, social, and educational differences between different populations, and these differences reflect in their QOL as a factor of increase or decrease vulnerability in the elderly. By categorizing the elderly into a single group of vulnerable people, one can incur the error of stereotyping this population, since the concept of vulnerability is considered by some authors as a label, or stereotype, not distinguishing individuals within this group (Luna, 2004). This consideration justifies the importance of individual analyses such as the questionnaire used in this research, which analyzes individuals within a group.

Considering gender as a factor, a phenomenon that accompanies population aging is the feminization of old age, however, despite living longer, women have a lower QOL due to the effect of gender relationships that structure social life and influence access to resources and opportunities, generating continuous and cumulative impacts on social and economic life (da Silva Sousa et al., 2018). A recent study on gender diversity found that gender-diverse Brazilian individuals represent around 2% of the country's adult population (almost 3 million people), and are homogeneously located throughout the country (Spizzirri et al., 2021), however, there are no official data on this population as they age, nor is it possible to make inferences from the data made available by Brazilian Institute of Geography and Statistics (IBGE) (Guimarães & Schramm, 2008).

Aging in Brazil occurs in a context of persistent inequality, and difficulties in the social protection and retirement system, however, to promote healthy aging, it is necessary to look at the elderly in an integral way considering all the variables that interfere with their QOL. According to data from IGBE of 2017, about 50 million Brazilians, which corresponds to 25.4% of the population, live in the poverty line (da Rocha, 2019). Also, according to IBGE, 2019 National Continuous Household Sample Survey (PNAD), the population of elderly in Brazil had the highest concentrations in the Southeast (17.1%) and in the South (17.4%). According to the same survey, the Northeast Region (11.9%) had the highest proportion of people declared black, followed by the Southeast (9.9%), Midwest (9.2%) and North (7.3%), with the Southern Region having a predominance of white population (73.2%)(IBGE - Intituto Brasileiro de Geografia e Estatistica, 2020). In a study conducted based on the 2008 PNAD, it was observed that black elderly had worse self-rated health compared to whites, however, regarding the inequality in mortality of the elderly according to race/color, little is known, possibly due to the problems observed in socioeconomic and statistical information in Brazil (Romero et al., 2019). What is currently available is that white elderly reach longer ages compared to others, with robust evidence of the disadvantages of the black and brown population in terms of living and health conditions(Romero et al., 2019).

The increase in the proportion of the elderly population raises a discussion about the needs of the elderly, and the breadth and complexity of the aging process (Barbosa et al., 2019). Aging can only be understood in its entirety. It is not only a biological fact but also a social and cultural fact (Freitas et al., 2010) and to age well, it is necessary to have QOL (H. A. Figueira et al., 2009). Potter stresses the importance of protecting the least privileged, the preservation of human dignity and social justice, and that all these needs call for social and inclusive ethics (Potter, 1999), this ethics as part of a Global Bioethics, where the global word was defined not only as global, but unified and comprehensive (Potter, 2000). Aging implies an increased risk for vulnerability, of a biological, socioeconomic, and psychosocial nature, and these conditions can impact the elderly, causing loss of QOL (Barbosa et al., 2017).

There are numerous factors that favor the promotion of QOL, among them social participation, social interaction, carrying out daily tasks with independence and autonomy, support and family contact as well as leisure activities (M. C. G. Ferreira et al., 2017). This statement is supported by our research, where high levels of QOL were found, with scores considered good and high among all age groups, in all facets of QOL self-analysis. Population in which 77.5% live with a family or spouse, with a higher QOL score among those who live with a spouse in relation to those who live with a family (*p* < 0.05). Some possibilities can explain these results, one of which would be that the elderly start to live like their family when beginning to have functional limitations and needs care or help from third parties, which could decrease his self-analysis of QOL. Another possibility may be related to the need for financial support from family members, since, in retirement, the elderly's income tends to be lower than when they are inserted in the labor market (Gvozd et al., 2019). Elderly have increased vulnerability due to the fragility of the social protection network, the asymmetry of the care relationship and the risk of harm to which they are subject in daily health care(Paranhos et al., 2017).

Lower QOL is associated with factors that increase vulnerability, such as extreme poverty and poor study conditions. In our study, we observed that those who declared lower income (1–2 minimum wages) had lower scores in the facet of the sensory functioning, in relation to those with higher income, which also decreased their total QOL score, but without statistical relevance in this study. A study carried out with a low-income population in Curitiba / Paraná reveals a prevalence of negative perception of health in elderly women with low income and low education, showing that worse socioeconomic conditions such as poor housing quality, economic dependence, or financial instability, directly contribute to a perception negative health status among the elderly (Vagetti et al., 2013). Potter believed that biomedical ethics needed to expand to include the responsibility of thinking and acting for the long term for the world population. He believed that only the bridge between medicine and ecology was not enough, and that the next phase of ethics should be the social justice (Potter, 2000). In addition to this condition, other dimensions must be considered in promoting the health of the elderly, such as, for example, the environment. According to WHO, the environment in which the elderly is inserted can determine dependence or not, being more likely that an elderly is physically and socially active if he can safely walk to his neighbors’ house, the park or use local transport. Physical limitations or disabilities usually influence the QOL of the elderly and are related to the loss of functionality (Rosalinn da Cruz et al., 2019).

Although aging can be understood as a non-pathological process, there is an inevitable progressive decrease in the functional reserve, which under conditions of overload can favor the installation of chronic pathological conditions. In the assessment of autonomy, we could observe good scores, with an average of 15.7 (78.5%), which demonstrates the maintenance of functionality in the studied group. In the age group >90 years, the lowest score was observed, suggesting a correlation between age and functional autonomy, which would be expected. In the functioning of the sensorium, we found high scores, with an average of 17.22 (86%) suggesting that the impairment of the sensorium does not interfere with the participants QOL. Two possibilities are raised, that the elderly in this group have not observed functional losses over time, or that such losses when present do not influence their well-being and QOL. Considering that the group studied had high educational e financial status, we could also conclude that the population studied has access to health care, and treatment possibilities for those losses when present.

The assessment of past, present, and future activity describes satisfaction with life achievements, future projects and desires, with the average obtained being above the average found in other studies, as a study that comparatively analyzed a group in Brazil (349 elderly) and in Portugal (508 elderly) where the average for this facet was 63.55% (Portugal) and 64.4% (Brazil) (Célia Ermel et al., 2017). A study carried out with 150 elderly in Paraíba / Brazil, who were institutionalized or awaiting institutionalization in a long-term care institution obtained an average percentage of 50.5% in this facet, being the lowest average found in the study (Araújo & Bós, 2017). Another study that analyzed the difference between long-term care institution residents and non-residents, in Bauru / São Paulo demonstrated that those who live in long term institutions have lower QOL, mainly in this aspect, which demonstrates a lack of perspective on the lives of asylum seekers, who no longer design activities and future interests (Simeão et al., 2018). The present study could observe that the participants have a good perception of the past and future planning with a score of 73%, and that it may be associated with the place and with whom they live, in addition to maintaining work and voluntary activities.

In addition to possible limitations in relation to carrying out activities of daily living, elderly face several obstacles, including attitudinal, environmental, and institutional barriers, which prevent their full and equal participation in all aspects of life (Martins et al., 2020). When evaluating social participation, this study could observe an average score of 14.99 (74.9%) which demonstrates that there is a loss of social participation within this group, although it is still a regular average, since other studies suggest a significant loss in social participation of the elderly(O. Figueira et al., 2020), while reinforcing the importance of social interaction as a measure to promote QOL. A study carried out with 1691 elderly in Minas Gerais / Brazil obtained 63.4% in the average percentage of the social participation facet, indicating that the elderly in that study found fewer opportunities to participate in activities in the community. Social isolation denotes the need for encouragement and support for activities by a multidisciplinary team, inserting the elderly in actions that make them feel valued and, thus, resulting in improved social relationships and self-esteem (Rodrigues et al., 2017).

WHO itself recognized social support as an important factor in promoting quality health and aging (Maia et al., 2016), which when present reduces the stigmatization of the elderly and, consequently prejudice. The discussion about prejudice, discrimination and stigma in aging is complex and cannot be generalized, as they are accentuated depending on the cultural and social context, together with the social representations that surround aging and that are reflected in the treatment of the elderly, demonstrating the importance of adopting actions to reframe aging and its nuances in society (Laynne et al., 2020).

A factor that increases the vulnerability of the elderly is being a victim of social and structural prejudices, in a negative current socio-cultural view on aging, due to misconceptions that currently exist, amplified by an overload of stereotypes and prejudices that do not correspond to reality inherent in human old age (Schwertner & Bodnar, 2019). It must be considered that stereotyping implies fixing a label on individuals or groups, which cannot be easily removed and can prevent us from identifying levels of vulnerability. In a way, the concept of vulnerability has stereotyped entire categories of individuals, without distinguishing between individuals in the group who may have special characteristics that need to be considered (Luna, 2019), which is why the self-analysis proposed by WHOQOL-Old is so important, for separating individuals within the same group.

Potter considers the importance of the social and cultural context in the QOL and recognizes them as paramount in the proposal of global bioethics for a more just world. Solidarity can be considered a proposal to achieve the socially just. Bioethics, since its conception, has prioritized respect for individual autonomy, and with that, it calls for themes beyond the individual, such as socio-political issues and the different relationships that subjects have in this context, including responsibilities, obligations, and claims. Bioethics, by embracing the concept of “solidarity” seeks to mobilize forces and resources for the less privileged segments of our society, as well as building community and legal guarantees of fundamental rights that guarantee a dignified and happy living (Pessini, 2017). It must be a practice and not just an inner feeling of abstract value, it brings the need for action, which all of us, as a society, must have in relation to the other, particularly the most vulnerable, it is associated with the rescue of citizenship and the fundamental rights of life. Beauvoir expresses that society tries to homogenize old age, describing it as an object, analyzed from the outside. She proposed that aging occurs within a society and therefore depends on the place that the subject occupies in it. For her, rejection of the elderly goes beyond self-defense, in the sense of avoiding confrontation with finitude, it also indicates the way in which society deals with the elderly, being defined by a global system of values (Furtado Nogueira & Bloc Boris, 2019).

The present study found in the facet death and dying the lowest overall average, of 14.33 (71.6%), which reduced the QOL, although it still qualified as regular, converging with other studies(Billett et al., 2019; Scherrer Júnior et al., 2020) where this relationship with death and dying is satisfactory, including a study carried out in support homes in Switzerland, where the results showed that fragile elderly have little or no fear of dying (Sandgren et al., 2020). This data differs from a study carried out in Santa Catarina / Brazil with 122 elderly, with incomplete primary education and a total QOL score of around 50%, with facet death and dying being the lowest score(Gato et al., 2018). The passage of time and the perception of aging itself can be a factor that generates anguish and uncertainty. For some individuals, old age means the proximity of death, which is why it generates fear, for others, it can have another meaning, and show itself as a possibility to enjoy life, and the remembrance about finitude itself indicatingthe importance of living (Ribeiro, 2019).

In the assessment of intimacy, the values found are good, with an average of 15.82 (79.1%) suggesting the satisfaction of the elderly in this regard. Study with 1691 elderly in Minas Gerais / Brazil, with the population aged 70–80 years (43.5%), married (43.1%), with 4–8 years of study (35.7%), income of a minimum monthly salary (47.8%) and living with their children (40.7%), found an intimacy score of 70%, values close to the present study, despite a different sociodemographic profile (Rodrigues et al., 2017). Sexuality is not directly addressed in the inventory, and during the WHOQOL-Old validation and formulation, questions related to it were removed, as it was observed that for the elderly, intimacy was more important and significant than sexual activity itself (OMS, 2005).

Bioethics was formulated by Potter to integrate biology and human values, especially moral philosophy. Since the publication of Potter in 1971 of the work “Bioethics: bridge to the future”, there has been an attempt to build bridges between knowledge, between practices and between society (Zanella et al., 2019). Now it is necessary to build a bridge between the vulnerability of the elderly and aging with dignity. This bridge will allow the elderly to leave the position of increased vulnerability achieving QOL in aging. Considering the multiple dimension approach to both the understanding of vulnerability and the proposal for healthy aging, it should be noted that Potter's bioethics proposal addresses the importance of interdisciplinarity. Potter mentions that humanity's problems are multidimensional and facing them implies that we have to relate different types of knowledge, from different disciplines, such as basic biology, social sciences and humanities (Pessini, 2019).

Despite the situation of vulnerability that is natural to them, and the increased risk of adding layers of vulnerability, which can be biological, socioeconomic, and psychosocial in nature, there seems to be an opposite movement of the elderly population to remain healthy throughout aging. This movement is supported by WHO when proposing the Decade of Healthy Aging (2021–2030) and by the proposal of Potter's global bioethics, which seeks to give voice to a broader view of ethics in relation to health, life and death, the society, and public policies. Potter recognizes that individual health and QOL are related to the health of society and culture, and that is why one cannot think of these aspects separately, but in an integrated way, since the illness of one of the parties harms the integral health of the individual (Sganzerla, 2020). In one of his last speeches in 2001, Potter declared that global bioethics must evolve into politically energized and socially concerned world bioethics, that this new bioethics for the twentieth century calls for care for people, health, land and with animals. Therefore it is so important to identify the different layers of vulnerabilities, making it possible to adopt measures that can remove these layers of vulnerability promoting healthy aging (Luna, 2019). To disregard the multiplicity of issues involving aging, such as social, cultural, economic factors, gender differences, race, etc. is to disregard aging process as it is, a complex, dynamic and multidimensional process.

## Conclusion

The population studied perceived their QOL positively, and analyzing factors associated with increased vulnerability in the WHOQOL-Old inventory, it was found that the facets of death and dying and social participation were those that reduced their QOL. To promote healthy aging from the perspective of Potteŕs global bioethics, it is necessary to broaden the vision for social and cultural justice, therefore it is important to recognize that social, cultural e economic differences can be an important factor of such good QOL levels found in this study. Potter recognizes that human QOL is related to the society and culture in which we operate, and it is up to us to propose a paradigm shift in relation to aging, with the aim of promoting a more just and humanly supportive society.

### Study limitations

As a study that analyzed elderly with access to technology and social media, with a postgraduate educational profile and a high income, its result cannot be generalized for the entire Brazilian population. Different contexts may present specificities not covered in the present analysis.

## References

[CIT0003] Araújo, A. M., & Bós, JÂG. (2017). Qualidade de vida da pessoa idosa conforme nível de institucionalização. *Estudos Interdisciplinares Sobre o Envelhecimento*, *22*(3), 3. 10.22456/2316-2171.60224

[CIT0004] Barbosa, K. T. F., de Freitas Macêdo Costa, K. N., de Farias Pontes, M. d. L., de Souza Batista, P. S., de Oliveira, F. M. R. L., & Fernandes, M. d. G. M. (2017). Aging and individual vulnerability: A panorama of older adults attended by the family health strategy. *Texto e Contexto Enfermagem*, *26*(2), 2700015. 10.1590/0104-07072017002700015

[CIT0005] Barbosa, K. T. F., Oliveira, F. M. R. L. d., & Fernandes, M. d. G. M. (2019). Vulnerability of the elderly: A conceptual analysis. *Revista Brasileira de Enfermagem*, *72*(2), 337–344. 10.1590/0034-7167-2018-0728 31826228

[CIT0006] Billett, M. C., Campanharo, C. R. V., Lopes, M. C. B. T., Batista, R. E. A., Belasco, A. G. S., & Okuno, M. F. P. (2019). Functional capacity and quality of life of hospitalized octogenarians. *Revista Brasileira de Enfermagem*, *72*(2), 43–48. 10.1590/0034-7167-2017-0781 31826190

[CIT0007] Célia Ermel, R., Caramelo, A. C., Fracolli, L. A., Boas, F. V., Ortiz, C., Lais, T., Zutin, M., Soares Gianini, S. H., Morelatto, L., Da Silva, P., Cardin, M. A., Guimarães, R., Zutin, P., Dos, J., Dorvalino, S., & Vieira, M. (2017). Percepção sobre qualidade de vida dos idosos de Portugal e do brasil. *Revista Eletrônica Acervo Saúde*, *9*(2), 1315–1320. 10.25248/REAS98_2017

[CIT0008] da Rocha, G. B. F. (2019). A importância das condições socioeconômicas na elaboração de políticas públicas voltadas à saúde do idoso. *Revista Longeviver*. Ano I, n. 3, Jul/Ago/Set, São Paulo. https://revistalongeviver.com.br/index.php/revistaportal/article/viewFile/788/843.

[CIT0009] da Silva Sousa, N. F., Lima, M. G., Cesar, C. L. G., & de Azevedo Barros, M. B. (2018). Envelhecimento ativo: Prevalência e diferenças de gênero e idade em estudo de base populacional. *Cadernos de Saúde Pública*, *34*(11), 78088–78225. 10.1590/0102-311X00173317 30484561

[CIT0010] Ferreira, M. C. G., Tura, L. F. R., da Silva, R. C., & Ferreira, M. d. A. (2017). Social representations of older adults regarding quality of life. *Revista Brasileira de Enfermagem*, *70*(4), 806–813. 10.1590/0034-7167-2017-0097 28793112

[CIT0011] Ferreira, V. H. S., Leão, L. R. B., & Faustino, A. M. (2020). Ageísmo, políticas públicas voltadas para população idosa e participação social. *Revista Eletrônica Acervo Saúde*, *42*(42), e2816–e2816. 10.25248/reas.e2816.2020

[CIT0012] Figueira, H. A., Figueira, J. A., Bezerra, J. C., & Dantas, E. H. M. (2009). Old aged quality of life: Brazil – India a cross-cultural perspective. *IndiaN Journal of Gerontology*, *23*(1), 66–78.

[CIT0013] Figueira, O., Figueira, H., Dantas, E. H. M., Soleiman Franco, R., & Corradi Perini, C. (2020). Estratégias para a promoção do envelhecimento ativo no brasil: Uma revisão integrativa. *Research, Society and Development*, *9*(10). e1959108556. 10.33448/rsd-v9i10.8556

[CIT0014] Fleck, M., Leal, O., & Louzada, S. M. X.-B. J. of, & 1999, U. (1999). Desenvolvimento da versão em português do instrumento de avaliação de qualidade de vida da OMS (WHOQOL-100). *Brazilian Journal of Psychiatry*, *21*(1), 19–28. 10.1590/S1516-44461999000100006

[CIT0015] Freitas, M. C. d., Queiroz, T. A., & Sousa, J. A. V. d. (2010). O significado da velhice e da experiência de envelhecer para os idosos. *Revista Da Escola de Enfermagem Da USP*, *44*(2), 407–412. 10.1590/S0080-62342010000200024 20642054

[CIT0016] Furtado Nogueira, C., & Bloc Boris, G. D. (2019). Envelhecimento na perspectiva fenomenológico-existencial de sartre e de beauvoir. *Revista de Psicología*, *28*(2), 1–14. 10.5354/0719-0581.2019.55661

[CIT0017] Gato, J. M., Zenevicz, L. T., Madureira, V. S. F., Silva, T. G. d., Celich, K. L. S., Souza, S. d., & Léo, M. M. F. d. (2018). Saúde mental e qualidade de vida de pessoas idosas. *Avances En Enfermería*, *36*(3), 302–310. 10.15446/av.enferm.v36n3.68498

[CIT0018] Guimarães, A., & Schramm, F. R. (2008). A Bioética da proteção e o envelhecimento da população transexual. *Revista Brasileira de Bioética*, *4*(1–2), 80–96.

[CIT0019] Gvozd, R., Rossaneis, M. A., De, P., Cavalcante, S., & Ii, P. (2019). Cultural adaptation of the retirement resources inventory for Brazilian culture. *Revista de Saúde Pública*, *53*, 60. 10.11606/s1518-8787.2019053000863 31340352 PMC6629292

[CIT0002] IBGE - Intituto Brasileiro de Geografia e Estatistica. (2020). Pesquisa Nacional por Amostra de Domicílios Contínua - PNAD. https://biblioteca.ibge.gov.br/.

[CIT0020] Laynne, I., Machado, O., & Garrafa, V. (2020). Bioética, o envelhecimento no Brasil e o dever do estado em garantir o respeito aos direitos fundamentais das pessoas idosas. *Revista De Direitos E Garantias Fundamentais*, *21*(1), 79–106. 10.18759/rdgf.v21i1.1804

[CIT0021] Lima-Costa, M. F., Andrade, F. B. d., Souza Jr, P. R. d., Neri, A. L., Duarte, Y. A. d. O., Castro-Costa, E., & Oliveira, C. d. (2018). The Brazilian longitudinal study of aging (ELSI-Brazil): objectives and design. *American Journal of Epidemiology*, *187*(7), 1345. 10.1093/aje/kwx387 29394304 PMC6031009

[CIT0022] Luna, F. (2004). Vulnerabilidad: La metáfora de las capas. *Journal of Bioethics*, *4*(3), 44–49. 10.1080/15265160490497083

[CIT0023] Luna, F. (2019). Identifying and evaluating layers of vulnerability–a way forward. *Developing World Bioethics*, *19*(2), 86–95. 10.1111/dewb.12206 30058768 PMC6353708

[CIT0024] Maia, C., Castro, F., … A. da, F.-R. I. (2016). Redes de apoio social e de suporte social e envelhecimento ativo. *International Journal of Developmental and Educational Psychology. Revista INFAD de Psicología*, *1*(1), 293–304. 10.17060/ijodaep.2016.n1.v1.279

[CIT0025] Martins, J. A., Watanabe, H. A. W., Braga, V. A. S., Jesus, M. C. P. d., & Merighi, M. A. B. (2020). Older adults with physical disabilities: Vulnerabilities relative to the body, the physical and social environment. *Revista Brasileira de Enfermagem*, *73*(Suppl 3), e20190175. 10.1590/0034-7167-2019-0175 32667414

[CIT0001] OMS. (2005). Whoqol-old, Manual da Organizaçao Mundial da Saude. https://www.who.int/mental_health/evidence/WHOQOL_OLD_Manual.pdf

[CIT0026] OMS, O. M. d. S. (2020). Década do Envelhecimento Saudável 2020–2030. https://www.paho.org/pt/decada-do-envelhecimento-saudavel-2020-2030

[CIT0027] Paranhos, D. G. A. M., Albuquerque, A., & Garrafa, V. (2017). Vulnerabilidade do paciente idoso à luz do princípio do cuidado centrado no paciente. *Saúde e Sociedade*, *26*(4), 932–942. 10.1590/s0104-12902017170187

[CIT0028] Pessini, L. (2017). Elementos para uma bioética global: Solidariedade, vulnerabilidade e precaução. *Thaumazein: Revista Online de Filosofia*, *10*(19), 75–85.

[CIT0029] Pessini, L. (2019). Bioetica global em tempos de incertezas, perplexidades e esperanças. https://www.camilianos.org.br/area/img/bioetica_global_ebook_FINAL_22fev2019.pdf

[CIT0030] Potter, V. R. (1999). Fragmented ethics and “bridge bioethics”. *The Hastings Center Report*, *29*(1), 38. 10.2307/3528538 10052010

[CIT0031] Potter, V. R. (2000). Global bioethics with humility and responsibility. *Biomedical Ethics*, *5*(2), 89–93. 11822423

[CIT0032] Potter, V. R., & Whitehouse, P. J. (1998). Deep and global bioethics for a livable third millennium. *The Scientist*, *12*(1), 9.

[CIT0033] Ribeiro, AÂV. (2019). O envelhecimento ativo e a “boa” morte. *Revista Brasileira de Sociologia Da Emoção*, *18*(53), 131–141. http://www.cchla.ufpb.br/rbse/Oenvelhecimentoativoea%22boa%22morte.

[CIT0034] Rodrigues, L. R., Tavares, D. S., Dias, F. A., Pegorari, M. S., Marchiori, G. F., & Tavares, D. M. d. S. (2017). Qualidade de vida de idosos comunitários e fatores associados. *Revista de Enfermagem UFPE on Line*, *11*(supl.3), 1430–1438.

[CIT0035] Romero, D. E., Maia, L., & Muzy, J. (2019). Tendência e desigualdade na completude da informação sobre raça/cor dos óbitos de idosos no sistema de informações sobre mortalidade no brasil, entre 2000 e 2015. *Cadernos de Saúde Pública*, *35*(12), e00223218. 10.1590/0102-311x00223218 31800792

[CIT0036] Rosalinn da Cruz, R., Beltrame, V., Meneghetti Dallacosta, F., & Rubia Rosalinn da Cruz, C. (2019). Aging and vulnerability: An analysis of 1,062 elderly persons. *Revista Brasileira de Geriatria e Gerontologia*, *22*(3). 10.1590/1981-22562019022.180212

[CIT0037] Rudnicka, E., Napierała, P., Podfigurna, A., Męczekalski, B., Smolarczyk, R., & Grymowicz, M. (2020). The world Health Organization (WHO) approach to healthy ageing. *Maturitas*, *139*, 6–11. 10.1016/j.maturitas.2020.05.018 32747042 PMC7250103

[CIT0038] Sandgren, A., Arnoldsson, L., Lagerholm, A., & Bökberg, C. (2020). Quality of life among frail older persons (65+ years) in nursing homes: A cross-sectional sudy. Nursing Open. 10.1002/nop2.739 PMC8046081 34482652

[CIT0039] Scherrer Júnior, G., Portela, O. T., Passos, K. G., Okuno, M. F. P., Mesquita, M. S., Alonso, A. C., Barbosa, D. A., & & Belasco, A. G. S. (2020). Percepção da qualidade de vida de idosos residentes em instituições de longa permanência privada. *Enfermagem Brasil*, *19*(1), 20. 10.33233/eb.v19i1.2754

[CIT0040] Schwertner, M. R., & Bodnar, R. (2019). Trapos que aconchegam: O envelhecimento feminino em lygia fagundes telles. *Estudos de Literatura Brasileira Contemporânea*, *56*. 10.1590/2316-4018569

[CIT0041] Scoralick-Lempke, N. N., Do Nascimento, E., Ribeiro, B. C. S., Moreira, C., Oliveira, M. E. L., Sousa, P. C., & Teixeira, T. J. (2018). Comportamentos de saúde e envelhecimento saudável: Um estudo com idosos da comunidade. *Revista Família, Ciclos de Vida e Saúde No Contexto Social*, *6*(4), 775–784. 10.18554/refacs.v6i4.3293

[CIT0042] Sganzerla, A. (2020). Hans Jonas e a promoção da Bioética global de VR Potter. *Pensando-Revista de Filosofia*, *11*(24), 24. 10.26694/pensando.v11i24.11390

[CIT0043] Simeão, S. F. d. A. P., Martins, G. A. d. L., Gatti, M. A. N., Conti, M. H. S. D., Vitta, A. D., & Marta, S. N. (2018). Estudo comparativo da qualidade de vida de idosos asilados e frequentadores do centro dia. *Ciência & Saúde Coletiva*, *23*(11), 3923–3934. 10.1590/1413-812320182311.21742016 30427462

[CIT0044] Spizzirri, G., Eufrásio, R., Lima, M. C. P., de Carvalho Nunes, H. R., Kreukels, B. P. C., Steensma, T. D., & Abdo, C. H. N. (2021). Proportion of people identified as transgender and non-binary gender in Brazil. *Scientific Reports*, *11*(1), 1–7. 10.1038/s41598-021-81411-4 33500432 PMC7838397

[CIT0045] Vagetti, G. C., Moreira, N. B., Filho, V. C. B., de Oliveira, V., Cancian, C. F., Mazzardo, O., & de Campos, W. (2013). Domínios da qualidade de vida associados à percepção de saúde: Um estudo com idosas de um programa de atividade física em bairros de baixa renda de Curitiba, Paraná, Brasil. *Ciência &amp; Saúde Coletiva*, *18*(12), 3483–3493. 10.1590/S1413-81232013001200005 24263865

[CIT0046] Zanella, D. C. (2018). Humanidades e ciência: Uma leitura a partir da Bioética de Van Rensselaer (V. R.) Potter. *Interface - Comunicação, Saúde, Educação*, *22*(65), 473–480. 10.1590/1807-57622016.0914

[CIT0047] Zanella, D. C., Sganzerla, A., & Pessini, L. (2019). VR Potter's global bioethics. *Ambiente &amp; Sociedade*, *22*, 2081. 10.1590/1809-4422asoc20180208r1vu2019l3rs

